# #NotTheSame: Asian American subgroups moderate the relation between campus racial climate and perceived burdensomeness during the COVID-19 pandemic

**DOI:** 10.3389/fpubh.2023.982535

**Published:** 2023-03-20

**Authors:** Joel Jin, Vanessa Zhou, Trevor Taone, Emi Ichimura

**Affiliations:** Department of Clinical Psychology, Seattle Pacific University, Seattle, WA, United States

**Keywords:** anti-Asian racism, campus racial climate, suicide risk, perceived burdensomeness, college mental health, disaggregate

## Abstract

The present study examined the effect of campus racial climate on perceived burdensomeness, a suicide risk factor, among Asian American college students during the COVID-19 pandemic, when anti-Asian racism was present. To disaggregate these data, there was a test of whether Asian American ethnicity subgroup identification as Southeast and South or East Asian changed the association between campus racial climate on perceived burdensomeness. The current sample included 148 college students, 73 Southeast or South Asian Americans, and 75 East Asian American. The study participants were enrolled at a small liberal arts institution located in the Pacific Northwest region of the United States. Researchers collected data across 3 days (9–12 April 2020) *via* an online questionnaire. Both groups reported similar levels of campus racial climate and perceived burdensomeness. Bivariate correlations indicated that campus racial climate was positively correlated with perceived burdensomeness for Southeast and South Asians only. Moderation analyses revealed that a negative campus racial climate was related to greater perceived burdensomeness among Southeast and South Asian, but not East Asian, American students. This finding supports the need for disaggregation of Asian subgroups in mental health research to understand the diverse experiences within the Asian American community. Furthermore, there is a need for higher education institutions to consider tailoring interventions and tools that fit into the unique cultural and sociohistorical experiences of ethnic and racial subgroups among Asian American students.

## 1. Introduction

The syndemic of COVID-19 and anti-Asian racism has compounded and exacerbated the negative effects of one another (1). Discrimination to the degree of hate crimes was rampant, and inequalities across health, social, and economic spheres were exposed (2, 3). Such discrimination is historical (4–7), systemically disadvantaging people of Asian heritage and perceiving them as a “threat” and a “perpetual foreigner,” but still harmful today (8–10). Due to the recent pandemic-related anti-Asian racism, there is a concern about the increase in negative mental health symptoms among Asian Americans (11, 12). Among Asian American (AsAm) college students during the pandemic, racial discrimination was associated with a greater likelihood of endorsing at least one clinically significant mental health condition, including suicidal ideation and moderately severe or severe depression (12). Prior to the pandemic, suicide was documented as the second leading cause of death for AsAm college students aged 15–34 years (13, 14), and AsAm college students were 1.6 times more likely to seriously consider suicide attempts than white college students (15). Together with the latest research on the increasing suicide attempts and ideation among AsAm college students during the pandemic (16, 17), this study will examine the impact of racism-related stressors on suicide risk factors among AsAm college students.

Of the myriad of stressors that AsAm students experience, racial discrimination is of note due to the link between discrimination and negative mental health symptoms (18, 19). Furthermore, the danger of suicide among AsAm young adults highlights the relationship between discrimination and suicide ideation or suicide risk factors, specifically perceived burdensomeness (20). Perceived discrimination predicting suicide risk is found among other racially minoritized groups as well (21). Thereby, researchers are becoming more cognizant of the ongoing, pervasive experience of prejudice and discrimination against minoritized people and its effects on mental wellbeing and risk factors. For this study, we examined perceived burdensomeness, a suicide risk factor, among AsAm college students.

Joiner (22) coined the term “interpersonal needs” to describe the two main psychological states that incite one's desire for death by suicide—perceived burdensomeness, the feeling of being a liability and burden to others, and thwarted belongingness, which is defined as one's presumed ineptitude to higher quality relationships. Although previous studies have tested this theory among predominantly white people, several studies endorse the use of this theory among minoritized populations as well. Within a sample of university students from China, for instance, researchers observed a significant relationship between interpersonal needs and suicide ideation while controlling for gender, social support, self-esteem, and age (23).

Given the cultural values of group cohesion and harmony among AsAm college students, researchers have examined the impact of unmet interpersonal needs on depressive symptoms (24, 25) and higher shame (26). Perceived burdensomeness has drawn attention as a double bind to AsAm students since it may increase the desire for suicide and decrease the willingness to seek professional mental health help among AsAm college students (27). Qualitative studies on suicide are congruent in that unfulfilled interpersonal expectations strongly influence suicide ideation (20).

However, there is a gap in the literature on how racism-related stressors predict suicide risk factors among AsAm young adults. Wong et al. (20) summarized key themes relating to suicide risk for AsAm college students as “unfulfilled interpersonal expectations.” One negative interpersonal interaction was rejection and racism by a dominant group. According to these findings, we believe that negative racial experiences influence suicide ideation in this sample. In addition, Wang et al. (28) found that perceived discrimination predicted suicide ideation only at higher levels of perceived burdensomeness and thwarted belongingness among Asian international college students. The interaction between perceived discrimination and suicide risk factors appears to lead to negative mental health symptoms. Although pandemic-related anti-Asian racism and interpersonal needs remain unstudied, the current literature provides support for these relationships.

Studies also suggest that these patterns are different across AsAm ethnic subgroups. For instance, research on help-seeking behavior reveals that East Asian immigrants avoided seeking mental health treatment due to cultural stigma, honor/shame culture, family pride, financial constraints, a sense of failure, and lack of knowledge and awareness regarding mental illnesses (29, 30). However, participants from Southeast and South Asian countries were hindered by structural barriers, such as inaccessibility, unavailability, and unaffordability of resources, as well as cultural barriers, including loss of face, colonial mentality, and acculturation (30). Regarding the impact of discrimination, the negative effects are different when comparing the AsAm ethnic subgroups of adults (31) or generation status among AsAm college students (32). These are two examples of how identifying disaggregating AsAm data, whether by ethnic subgroups or generational status, can elucidate future mental health illness prevention and intervention.

Since we examined the experiences of young adults and college students, this study focused on campus racial climate as a proxy for racism-related stressors. An institution's climate is a global evaluation of an institution by its members based on observations on various dimensions, including racial climate (33). Campus racial climate is a student's lived experience and perception of their institution with regard to racial dynamics and racism. Studies have found that students of color have more negative and hostile perceptions of the racial climate on college campuses than their white peers (34, 35). Furthermore, students of color report more frequent experiences of racial discrimination than white students (36).

There is limited research on the impact of campus racial climate on the mental health of AsAm college students. Existing research shows that perceived negative campus climate is related to higher levels of self-reported depression among AsAm college students (37). Perceived racial discrimination is also associated with higher psychological distress, suicide ideation, anxiety, and depression in AsAm and Latino college students (18). Furthermore, much of the current literature lacks a report on disaggregated data on AsAm students. Museus and Park (38) conducted a qualitative study of AsAm undergraduates and their experiences with racism during college. Although all AsAm students reported that racial hostility and vicarious racism (witnessing racist acts directed at people of color) contributed to a reduced sense of safety and a greater climate of fear on campus, Laotian and Vietnamese students expressed feelings of isolation due to an underrepresentation of their own or other AAPI ethnic subgroups on campus. One mixed-method research study found that Filipino and Southeast Asian students reported the highest frequency of negative comments about race or ethnicity compared to East Asian and South Asian students (39). However, this finding was not statistically significant. The same study found that, compared to East Asians students, Southeast Asian students reported significantly greater dissatisfaction with their overall academic and social experiences. More studies are needed to understand the unique impact of racial climate on the mental health of students who belong to different ethnic and regional AAPI subgroups.

The aims of the present study are threefold: to disaggregate AsAm college student data to identify meaningful subgroup differences, to measure anti-Asian racism by college students' perception of their campus racial climate, and to examine detrimental effects of anti-Asian racism on perceived burdensomeness, which is a suicide risk factor. Thus, the study bridged the gap on the effects of campus racial climate on mental health risk factors and explored the effects of disaggregating AsAm college student data. The particularities of Asian ethnic subgroups were studied following an appeal for disaggregating data about AsAm health (40, 41), suicidology (42), and higher education (43). Specifically, we hypothesized that a negative campus racial climate would be associated with greater perceived discrimination, as we expect campus racial climate to function as a proxy for perceived racial discrimination on campus. We hypothesized that subgroup identification as Southeast and South Asians or East Asian would strengthen the association of campus racial climate on perceived burdensomeness. In other words, the campus racial climate during the COVID-19 pandemic would be associated with greater perceived burdensomeness depending on Asian ethnic subgroup identification.

## 2. Materials and methods

### 2.1. Participants

A total of 158 Asian American (AsAm) college students participated in the larger project. Researchers categorized 73 participants as Southeast/South Asian (Filipino, Vietnamese, Indian, Thai, Pakistani, Burmese, Cambodian, Indonesian, Singaporean), 75 as East Asian (Chinese, Korean, Taiwanese, Japanese), eight as multiethnic (denoting two or more ethnicities such as Chinese and Filipino), one as Native Hawaiian and Pacific Islander, and one where the open-ended ethnicity self-identification question was left blank. We included only college students of Southeast/South and East Asian ethnicity for the current study (*n* = 148) and excluded participants categorized as multiethnic or without specific ethnic information.

### 2.2. Procedure

The present study received the approval of the institutional review board. This study was part of a larger project that examined the perceptions of institutional support among AsAm college students (44) and the level of ethnocultural empathy toward Asian and AsAm college students by white college students (45) during the COVID-19 pandemic. Participants were eligible for the study if they were at least 18 years old and were enrolled in the university's undergraduate program. We collected data through an online, de-identified survey method over 3 days (9 April 2020–12 April 2020). Of note, the institution shifted to online learning on 13 April 2020. Participants volunteered to complete a brief survey. They were asked to provide non-documented informed consent, including a brief description of the project. At the end of the survey, participants could enter a raffle to win a $250 gift card for their participation. A debrief message expressed gratitude for their participation and provided participants with a list of resources for professional psychological help.

### 2.3. Measures

#### 2.3.1. Campus racial climate

We chose four of five items of the Racial Experiences subscale from Reid and Radhakrishnan (33) to measure campus racial climate (α = 0.70). We added a stem (e.g., “Since the COVID-19 outbreak…”) to each item to focus on reports of campus racial climate from the recent COVID-19 pandemic. Two of the sample items were “Since the COVID-19 outbreak, I have experienced racial insensitivity from other students” and “Since the COVID 19 outbreak, the interracial climate at Seattle Pacific University is tense.” Participants rated each item on a 7-point Likert scale (1 = *strongly disagree;* 7 = *strongly agree*). We used the average of four items. The measure demonstrated good internal consistency (α = 0.85).

#### 2.3.2. Perceived burdensomeness

A total of 10 items of the Interpersonal Needs Questionnaire (46) measured perceived burdensomeness (α = 0.89). Participants rated each item on a 7-point Likert scale (1 = *strongly disagree*, 7 = *strongly agree*). A sample item is “These days the people in my life would be better off if I were gone.” We used the average of all items. The measure demonstrated strong internal consistency (α = 0.90).

#### 2.3.3. Asian subgroup

We created a dichotomous variable to categorize our sample into two groups—East AsAm (i.e., referent group) or Southeast/South AsAm. Categorization was based on participant reports on an open-ended ethnicity self-identification question.

### 2.4. Statistical analysis

We conducted two main analyses. We ran independent-sample t-tests to compare the means of campus racial climate and perceived burdensomeness between East AsAm and Southeast/South AsAm groups. Then, we conducted a moderation using model 1 of the PROCESS macro in SPSS v.28 to examine whether the association between campus racial climate and perceived burdensomeness differed among East AsAm or Southeast/South AsAm groups. We included demographic variables that were significantly correlated with the dependent variable as confounding variables to the moderation.

## 3. Results

### 3.1. Descriptive statistics

The sample for this study consisted of 148 (101 women, 42 men, three non-binary, and two who preferred not to say) undergraduate students aged between 18 and 40 years (*M* = 20.60, *SD* = 3.22). Participants were from a small, predominantly white, Christian, liberal arts institution in the Pacific Northwest region of the United States. Approximately 18.8% of the undergraduate population was of Asian heritage at this predominately white institution, according to demographic information for the 2019–2020 academic year. The means and standard deviations of campus racial climate and perceived burdensomeness are presented in Table 1. Independent-sample *t*-tests supported no significant group differences between Southeast/South and East Asian groups on reports of campus racial climate (*t*_(146)_ = −0.123, *p* = 0.902) or perceived burdensomeness (*t*_(146)_ = −0.645, *p* = 0.520). The Pearson correlation coefficients demonstrated that campus racial climate was positively correlated with perceived burdensomeness (*r* = 0.267, *p* < 0.001) across the sample. Perceived burdensomeness was positively correlated with gender (*r* = 0.242, *p* < 0.01), but not with age (*r* = −0.177, *p* = 0.086). Among each subgroup, campus racial climate was positively correlated with perceived burdensomeness among Southeast/South Asians (*r* = 0.448, *p* < 0.001) but not among East Asians (*r* = 0.093, *p* = 0.426).

**Table 1:** Means and standard deviations by Asian subgroup and total sample.

	**Southeast/South Asian (*n* = 73) *M* (*SD*)**	**East Asian (*n* = 75) *M* (*SD*)**	**Total sample (*n* = 138) *M* (*SD*)**
Campus racial climate	2.90^a^ (1.38)	2.88^a^ (1.34)	2.89 (1.36)
Perceived burdensomeness	2.65^a^ (1.01)	2.54^a^ (1.05)	2.60 (1.03)

Means with similar superscripts denote means are not statistically different.

### 3.2. Moderation analysis

**Figure 1:**
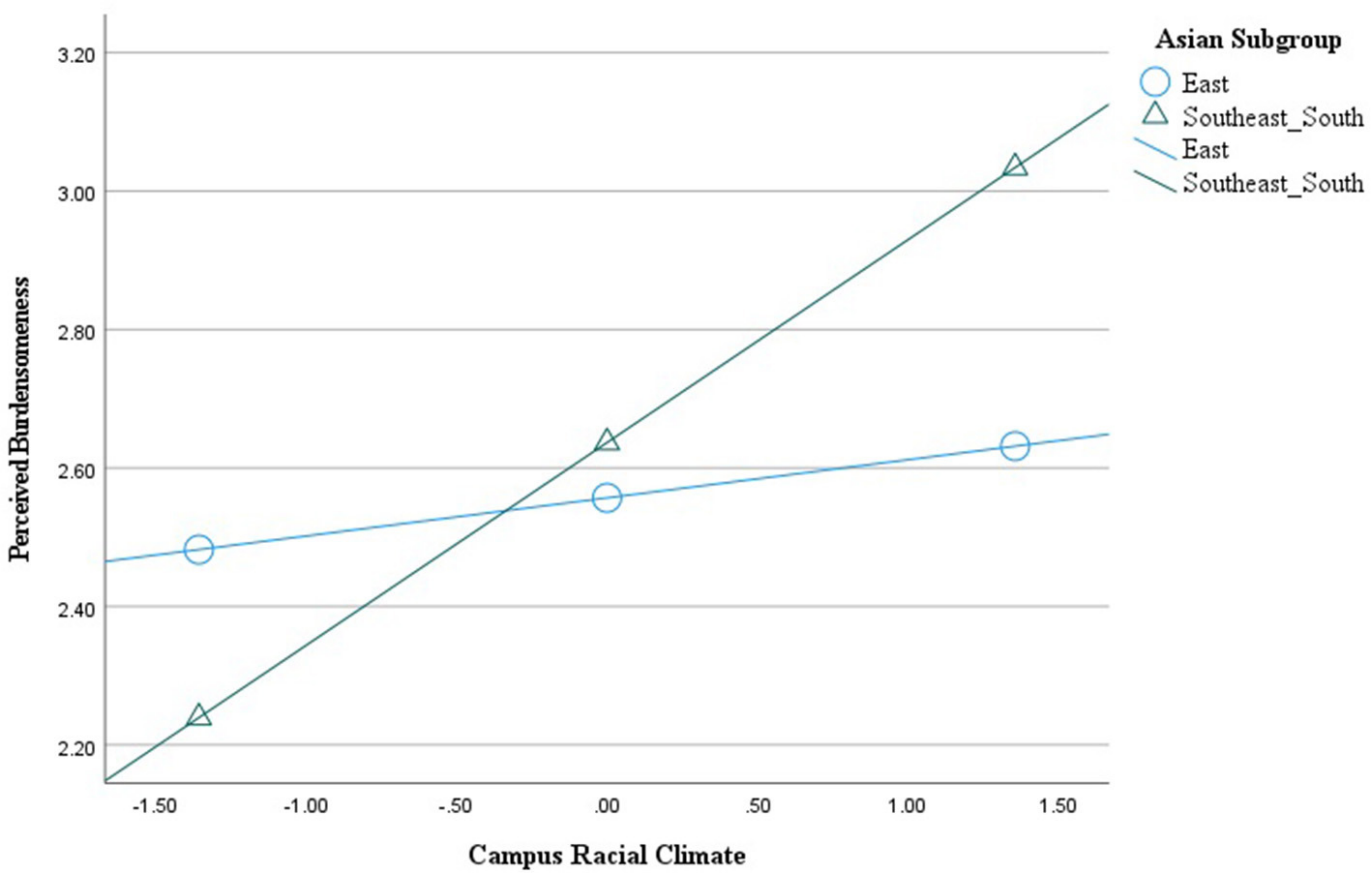
Asian subgroup interacting with the effect of campus racial climate on perceived burdensomeness.

We used model 1 of the PROCESS macro (47) in SPSS v.28 to test the moderation. We used 10,000 bootstrapped samples and mean-centered only the predictor (i.e., an observed, continuous variable campus racial climate) to construct the interaction term with the moderator (i.e., a dichotomous variable Asian subgroup). We identified the Asian subgroup as the moderator to test the effects of campus racial climate on perceived burdensomeness for each subgroup since this ethnic identification is a stable characteristic of participants that precedes their report of campus racial climate (48–50). As shown in Table 2, neither campus racial climate (*b* = 0.06, *t* = 0.65, *p* = 0.515) nor the Asian subgroup (*b* = 0.08, *t* = 0.50, *p* = 0.618) significantly predicted perceived burdensomeness. However, gender (*b* = 0.33, *t* = 2.36, *p* = 0.020) as a confounding variable significantly predicted perceived burdensomeness. Moreover, the interaction term did (Δ*R*^2^ = 0.03, *b* = 0.24, *t* = 2.01, *p* < 0.05), with a small effect size as well. Specifically, campus racial climate was significantly associated with greater perceived burdensomeness among Southeast/South Asians (*b* = 0.29, *t* = 3.48, *p* < 0.001) but not among East Asians (*b* = 0.06, *t* = 0.65, *p* = 0.515). Figure 1 displays the interaction effect.

**Table 2:** Interaction of Asian subgroup and campus racial climate on perceived burdensomeness.

**Variables**	***B***	**SE**	**95% CI**	***t***
Constant	1.97^***^	0.27	1.44, 2.50	7.38
Gender	0.33	0.14	0.05, 0.61	2.36
Campus Racial Climate	0.06	0.08	−0.11, 0.22	0.65
Asian subgroup (0 = East)	0.08	0.16	−0.24, 0.40	0.50
Racial climate × Asian subgroup	0.24^*^	0.12	0.00, 0.47	2.01

R^2^ = 0.14^***^.

CI, confidence interval based on 10,000 bootstrapped estimates.

* *p* < 0.05.

*** *p* < 0.001.

## 4. Discussion

The present study is one of the few to focus on the impact of campus racial climate on the mental health of AsAm college students. Given the increase in anti-Asian racism during the COVID-19 pandemic and the prevalence of suicide among AsAm young adults, we examined how perceived campus racial climate during the pandemic was associated with a suicide risk factor among AsAm college students. Contrary to our hypothesis, campus racial climate was not associated with perceived burdensomeness across the whole sample. Furthermore, the study empirically tested the interaction effect of Asian ethnic subgroup identification and campus racial climate on perceived burdensomeness. We found that campus racial climate was associated with greater perceived burdensomeness, a suicide risk factor, only among Southeast and South AsAm undergraduate students. There was no association between the two variables among AsAm students categorized as East Asian. The results provide empirical evidence that disaggregation of AsAm data was significant in understanding the detrimental effects of anti-Asian racism on the mental health of college students.

When conceptualizing campus racial climate as a report of racial discrimination, this study adds to the existing literature on the negative effects of perceived discrimination among AsAm students and the significance of suicide risk in this population. Moreover, this relationship was uncovered only when examining Asian ethnic subgroups compared to an aggregated AsAm sample. This is significant since perceived burdensomeness is a double bind for AsAm, a factor that both increases the desire for suicide and decreases the willingness to seek professional mental health help (31). This finding is contradictory to a previous study where no statistical difference among subgroups served as a justification to combine all Asian subgroups together (51).

Among AsAm college students categorized as Southeast and South Asian, our results converge with previous literature on pernicious racial experiences. Moreover, the results strengthen the association between discrimination with perceived burdensomeness in general [e.g., other studies have found similar effects of racial microaggressions among African Americans (52) and weight-based discrimination among community-dwelling adults (53)]. Park et al. (19) found that perceived discrimination was more impactful on South Asian college students' adjustment than East Asians. This is similar to findings among AsAm adults, for whom perceived discrimination is negatively correlated to life satisfaction only among Vietnamese and Asian Indians (31) and for Filipinos who report higher levels of discrimination than Chinese and Vietnamese adults (54). Though discrimination is not exclusive to certain Asian ethnic subgroups, there is evidence of differences.

However, there is a need to further explore the reasons for these differences. Park et al. (19) theorized that South Asian students are more impacted by perceived discrimination, possibly due to greater racial profiling based on phenotypical characteristics. The intersection of racism and race-based discrimination, along with colorism and skin color-based discrimination, may be the reason why Southeast and South Asian students are targeted (55). The overlapping systems of racism and colorism that oppress these AsAm students based on race and skin color may underlie the negative racial experiences they perceive on campus. Beyond everyday discrimination, Southeast and South Asians have a complex and diverse history of migration and trauma, including war, colonization, displacement, and resettlement [e.g., the Vietnam War and colonization in the Philippines (54)]. Finally, Huynh (56) analyzed a distinct domain of interethnic and intraethnic racism among second-generation Vietnamese in Southern California based on “presumed hierarchies of desirability” (p. 135). Overall, the detriment of a poor campus racial climate appears salient among these students due to the various types of racism that impact their lived experiences. As a result, it appears that negative racial experiences on campus, which are often interpersonal experiences with other students and faculty, can lead to viewing oneself as a burden on others.

Campus racial climate did not predict perceived burdensomeness among East Asian students in this study, even though Asian ethnic subgroups reported similar levels of campus racial climate and perceived burdensomeness. We hypothesized that this association would be significant due to Sinophobia and the return of the “yellow peril” (57, 58). It is unclear why this might be the case, and there is limited literature on these associations using disaggregated sample data gathered from AsAm students. Here, we offer a few theoretical conceptualizations and limitations of this study as possible explanations. The disconnect between negative racial experiences on campus and perceived burdensomeness among East Asians could be related to differences in racialized backgrounds. For instance, Museus and Truong (43) examined the perceptions of campus racial climate among AsAm college students from predominantly white high schools and predominantly racial minority high schools. Qualitative analyses demonstrated that students from predominantly white high schools reported more positive appraisals of campus climate, less stress due to racial prejudice and discrimination, and tended to downplay or rationalize racial stereotypes. Asian ethnic subgroup information was not provided to study participants, yet this supports the notion that high school background is one factor underlying the complex and diverse lived experience of AsAm students. Other protective factors of racism-related stress include older generation status (32) and both independent and interdependent self-construal (20). Future studies could examine these possible protective factors. There are limitations of this study as well. Of note is the use of campus racial climate as the only measure of racism-related stressors and racial discrimination. Since there are many forms of racism that were not observed in this study, perhaps other types of racism would predict perceived burdensomeness. Overall, there is a need for further investigation on the impact of campus racial climate among AsAm students whether across Asian ethnic subgroups or within ethnic subgroups.

Having interpreted the results of this study, we acknowledge the limitations. First, we categorized students of Southeast and South Asian ethnicities into one group, though our study promotes disaggregation of data. Due to limitations in recruitment and sample size, we focused on contrasting East Asians with Southeast and South Asians for this study. Future research could target recruitment to maximize each subgroup and illuminate greater complexity and diversity within the AsAm population. Second, only undergraduate students were included in this study. Since graduate students could also be in the young adult age group and thus have a high risk for taking their own life, follow-up studies could be significant to understand how campus racial climate impacts them. Next, the sample was unrepresentative of AsAm students across higher education in the United States. Due to the sample size and institutional characteristics, these findings are limited in generalizability. The effects of campus racial climate among AsAm college students in other institutional settings may be different if AsAm students are racially minoritized or in the majorotiy. Fourth, we were unable to posit causal relationships since our study was cross-sectional. Data before and after the pandemic could be interesting to look at changes in levels of campus racial climate and perceived burdensomeness, especially related to how institutions and leadership intervene. Nevertheless, we considered Asian ethnic subgroup identification as the moderator in our model due to its stable and consistent quality as opposed to the campus racial climate. Finally, we only measured campus racial climate without considering its additive effect on top of the general campus climate. Future studies could clarify the unique effect of campus racial climate, above and beyond general campus climate, on mental health risk factors.

Despite these limitations, we identify the implications of our study. There is a need for higher education institutions to consider tailoring interventions and tools that fit into the heterogeneous cultural, historical, and sociopolitical experiences of ethnic and regional subgroups among AsAm students. In developing campus interventions for students, it is important to disaggregate data from AsAm students and consider differing immigration patterns among AsAm regional subgroups, such as among the commonly designated subgroups of East Asian, Southeast Asian, South Asian, and Filipino (59). For example, forced migration among refugees within the Southeast Asian communities or the impact of Spanish and U.S. colonialism on Filipino Americans has led to unique experiences with race and racism among these subgroups (59). Furthermore, it is imperative to create targeted interventions—such as a revised curriculum, cultural competency training, and campus events—that acknowledge the distinct racialized campus experiences between AsAm students from different regional subgroups. In addition, the creation of AsAm ethnic or regional subgroup-specific student organizations and institutional spaces could help improve the racial campus climate for these students and serve as a buffer against mental health difficulties (60, 61). Institutions are strongly urged to allot additional resources or partner with other universities to create supportive spaces for AsAm students who face barriers to accessing shared ethnic communities due to lower campus representation. Finally, it is important to consider how ethnic identity intersects with other dimensions of identity (e.g., gender, sexuality, class, and immigration status) among AsAm students to influence their racialized campus experience and mental health (42, 59). Institutional resources and support are crucial for AsAm college students to cocreate counterspaces where they can tell their counterstories (56) as active authors of their lives amid anti-Asian racism. Here, they could feel as though they are not a burden to others but could help empower advocacy for one another and themselves (62).

In summary, the present study provides empirical support for and adds to the existing literature on disaggregating Asian American data. Campus racial climate was associated with perceived burdensomeness, a suicide risk factor, for Southeast and South Asian American college students but not for East Asian American students. This interaction effect was found even though there were no group differences in these outcomes. Therefore, we acknowledge the complexity and diversity across Asian American communities and that we are #notthesame.

## Data availability statement

The raw data supporting the conclusions of this article will be made available by the authors, without undue reservation.

## Ethics statement

The studies involving human participants were reviewed and approved by Peter Rivera, PhD; Seattle Pacific University. The patients/participants provided their written informed consent to participate in this study.

## Author contributions

JJ: conceptualization, methodology, investigation, formal analysis, original draft, review, editing, and supervision. VZ: data curation, formal analysis, investigation, original draft, review, and editing. TT: data curation, formal analysis, visualization, and original draft. EI: interpretation of data and an original draft. All authors provided substantial contributions to the study, drafted critical content, provided final approval, and agreed to be accountable for the study.
